# 2472: Subjective cognitive complaints in mild traumatic brain injury and 6-month return to work prediction: A TRACK-TBI Study

**DOI:** 10.1017/cts.2017.112

**Published:** 2018-05-10

**Authors:** Debra Phillips, Laura Ngwenya, Michael Huang, Oi Saeng Hong

**Affiliations:** 1 University of California San Francisco, San Francisco, CA, USA

## Abstract

OBJECTIVES/SPECIFIC AIMS: About 75% of the estimated 2.5 million traumatic brain injuries (TBIs) diagnosed annually classify as mild TBI (mTBI); yet cognitive impairments associated with poor patient outcomes can persist for weeks to years. mTBI symptoms are difficult to measure objectively and often remain undiagnosed in the context of an unknown cognitive baseline. Formal neuropsychological exams hold limited utility due to their extensive resource burden. We aimed to define the clinical importance of a 4-question assessment of subjective cognitive complaints (SCC) in predicting return to work at 6 months following mTBI. METHODS/STUDY POPULATION: mTBI participants from the prospective Transforming Research and Clinical Knowledge in Traumatic Brain Injury Pilot Study were included. A self-report affirmation to at least 1 of 4 subjective cognitive symptoms yielded positive SCC. Regression analysis was used to determine factors associated with return to work by 6-months. RESULTS/ANTICIPATED RESULTS: Of 479 enrolled participants with mTBI, 271 (57%) had complete follow-up data. Of which, 156 (58%) had at least sheltered employment at enrollment. Thirty-four (22%) of workers had no return to work at 6-months. Demographics, prior education, presenting injury severity, work status, and post-traumatic stress disorder were associated with return to work. SCC was associated with lower odds of return to work by 6-months (OR=0.11, *p*=0.01). DISCUSSION/SIGNIFICANCE OF IMPACT: We suggest a concise 4-question assessment of SCC may be clinically relevant in estimating the likelihood of return to work by 6 months post-mTBI.

